# Impact of *Staphylococcus aureus* protein A (spa) genetic typing in cases of prosthetic shunt graft infections

**DOI:** 10.1007/s00772-016-0146-6

**Published:** 2016-06-20

**Authors:** P. Konstantiniuk, A. Grisold, G. Schramayer, S. C. Santler, S. Koter, T. Cohnert

**Affiliations:** 1 Department of Surgery, Division of Vascular Surgery, Medical University of Graz, Auenbruggerplatz 29, 8036 Graz, Austria; 2 Institute of Hygiene, Microbiology and Environmental Medicine, Medical University of Graz, Graz, Austria

**Keywords:** Shunt graft, Infection, *Staphylococcus aureus*, Spa typing, Nosocomial, Shuntprothese, Infektion, *Staphylococcus aureus*, Spa-Typisierung, Nosokomial

## Abstract

**Introduction:**

In January 2014 an internal audit was performed at the department of surgery, division of vascular surgery of the Medical University Graz, Austria, to assess the short and long-term outcomes of prosthetic shunt graft implantations performed between December 1998 and December 2013. A 10.8 % explantation rate due to graft infection was detected. The majority of the cases were associated with *Staphylococcus aureus*. The aim of this study was to clarify whether this constitutes a nosocomial problem.

**Patients and methods:**

Between December 1998 and December 2014 a total of 490 prosthetic shunt grafts were implanted. After exclusion of 54 cases, 436 shunts remained for statistical analysis. Genetic analysis (spa typing) was acquired from three new cases with involvement of *S. aureus* in 2014. The impact of several factors (e.g. sex, institute for dialysis, age, type of prosthesis, implantation surgeon and position of shunt) on the shunt graft infection rate was statistically analyzed.

**Results:**

Of the prostheses 14.0 % (61 out of 436) had to be explanted of which 12.4 % (54/436) were due to infection. In 77.8 % (42/54) bacteria were found in blood and/or wound cultures. *Staphylococcus aureus* was present in 76.2 % (32/42) of the cases with detected bacteria and in all cases was sensitive to methicillin. The infection rate was not significantly dependent on any of the investigated factors; however, the factor “institute for dialysis” had a remarkable p‑value of 0.060 with the infection rate ranging from 8.5 % to 18.2 % depending on the institution. Three different *S. aureus* protein A (spa) types were found: t015, t359, t6265. The detection of three different spa types means that these patients had different sources of *S. aureus* so that a nosocomial problem is very unlikely.

**Conclusion:**

Genetic typing of spa is a suitable technique for distinguishing between nosocomial and community acquired sources of prosthetic shunt graft infections.

## Zusammenfassung

**Einleitung:**

Im Januar 2014 wurde an der Universitätsklinik für Chirurgie, Klinische Abteilung für Gefäßchirurgie, Medizinische Universität Graz, Österreich, ein internes audit über kurz- und langfristige Ergebnisse der im Zeitraum Dezember 1998 bis Dezember 2013 implantierten Shuntprothesen durchgeführt. Eine 10,8 %ige Explantationsrate aufgrund einer Infektion der Transplantate wurde festgestellt. Die Mehrzahl der Fälle war mit *Staphylococcus aureus* assoziiert. Das Ziel unserer Studie war es, zu klären, ob ein nosokomiales Problem vorliegt oder nicht.

**Patienten und Methoden:**

Zwischen Dezember 1998 und Dezember 2014 wurden 490 Shuntprothesen implantiert. Nach Ausschluss von 54 Fällen blieben 436 Shunts für die statistische Analyse. Drei neue Fälle aus dem Jahr 2014 mit Beteiligung von *S. aureus* konnten genetisch analysiert werden (spa-Typisierung). Der Einfluss verschiedener Faktoren (Geschlecht, Dialysezentrum, Alter, Art der Prothese, Chirurg der Implantation, Position des Shunt) auf die Shuntinfektionsrate wurde statistisch ausgewertet.

**Ergebnisse:**

Ein Anteil von 14,0 % (61/436) der Prothesen musste explantiert werden, 12,4 % (54/436) aufgrund einer Infektion. In 77,8 % (42/54) dieser Fälle wurden Bakterien in Blut- und/oder Wundkulturen gefunden. *S. aureus* wurde in 76,2 % (32/42) der Fälle mit positiver Bakterienkultur nachgewiesen, in allen Fällen war eine Empfindlichkeit gegenüber Methicillin vorhanden. Die Infektionsrate war nicht signifikant abhängig von den untersuchten Faktoren, jedoch hatte der Faktor „Dialysezentrum“ einen bemerkenswerten p‑Wert von 0,060 mit einem Infektionsratenbereich von 8,5 bis 18,2 % pro Einrichtung. Es wurden drei verschiedene *S. aureus* spa-Typen gefunden: t015, t359, t6265. Drei ungleiche spa-Typen bedeuten, dass diese Patienten verschiedene *S. aureus* Quellen hatten, so dass ein nosokomiales Problem sehr unwahrscheinlich ist.

**Schlussfolgerung:**

Die spa-Typisierung ist eine geeignete Technik, um zwischen nosokomialen und ambulant erworbenen Shunttransplantatinfektionen zu unterscheiden.

## Introduction

For hemodialysis of patients with renal failure, vascular access is needed. This is mainly achieved with a central venous catheter or by creation of an arteriovenous shunt. Best results for the latter in terms of functional duration and complication rates (infections, thrombosis) are gained by using autologous veins [1, 2]. In the absence of a suitable vein, various homologous, heterologous or prosthetic grafts are used [3]. At our institution, mainly expanded polytetrafluoroethylene (ePTFE) prostheses have been implanted for more than 15 years. Stenosis and thrombosis are frequent but can be corrected in the majority of cases by surgical thrombectomy and/or transluminal venous angioplasty [4, 5]. The most serious factor is bacterial infection, which is associated with high morbidity and mortality [6–9]. Gram-positive bacteria are the main cause of prosthetic shunt graft infections and *Staphylococcus aureus* is reported to be present in 60 % [10] of cases. Major complications (e.g. death or septic embolization to bones, joints, endocardium and brain) are found in 12 % of infected grafts [10]. Many microorganisms can switch from a free-living state to a sessile mode of life, forming a biofilm on surfaces [11]. The biofilm is a polymer matrix consisting of polysaccharides, proteins and DNA, which embeds the bacteria. This environment shows increased tolerance to antibiotics and resistance to phagocytosis and other components of the body’s defence system [12]. Shunt graft infections are therefore difficult to treat and so far, complete shunt graft explantation as early as possible is the treatment of choice.

In January 2014 we performed an internal audit on prosthetic shunt graft infections carried out between December 1998 and December 2013 and found a 10.8 % explantation rate due to infection. In 74.3 % of cases *S. aureus* was involved. Serious concern arose about the question whether there was a nosocomial problem. We decided to perform a detailed assessment and to include genetic techniques for upcoming shunt graft infections.

## Patients and methods

Patients with implantation of a PTFE shunt graft between 1 December 1998 and 31 December 2014 were included in the study. Only primary shunt grafts from artery to vein were included, short shunt grafts for corrective operations, such as interpositions were excluded. Infections of secondary shunt grafts from arteries to old prostheses or from prostheses to vein were categorized as primary shunt operation complications. Microbiological specimens included blood cultures and/or wound swabs. All shunt grafts were completely explanted. In most cases, a venous patch-plasty at the site of the arterial anastomosis and direct closure at the site of the venous anastomosis were performed. After explantation, open wound management was carried out, sometimes with negative pressure wound therapy. After achieving sufficient granulation on a clean surface, secondary wound closure was executed. Follow-up was registered until 28 February 2015. Data were acquired retrospectively from written reports in the hospital data system, from the patients and from the institutes performing dialysis. In 2014, three cases with *S. aureus* underwent genetic examination using the spa typing method. Protein A is a surface protein of the cell wall of *S. aureus*. It is encoded by the *spa* gene which can be typed on the basis of the base sequence. Spa types are recorded and provided by a worldwide usable server project (Ridom SpaServer [13]). Up to 12 January 2016, a total of 15,573 different spa types were recorded by 668 registered users from 60 user countries (117 strain countries). Influencing factors were assessed with χ^2^-test, Fisher’s exact test or logistic regression using IBM® SPSS® Statistics 23.0.0.0. All tests were two-sided and P‑values below 0.05 were regarded as significant.

## Results

**Fig. 1:**
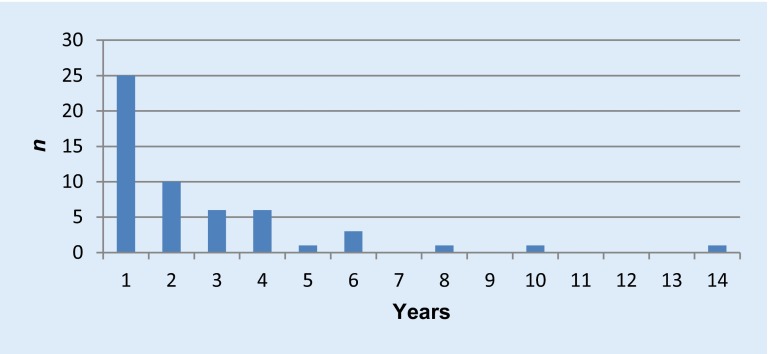
Number of PTFE shunt graft explantations due to infection itemized in years after the implantation (totalling 54)

From 1 December 1998 to 31 December 2014, a total of 1431 shunt operations were performed at the Department of Surgery, Division of Vascular Surgery, MUG, Austria, 490 of which were PTFE shunt graft implantations (shunt graft rate 34.2 %). In total 54 cases had to be excluded due to the exclusion criteria, 42.2 % (184/436) had a carbonized inner surface (Venaflo®, Carboflo®, C.R. Bard, Inc. Corporate Office, Murray Hill, New Jersey, USA), 20 % (87/437) were plain PTFE (Gore-Tex® vascular graft, standard walled or Gore-Tex® vascular graft, thin-walled ringed or unringed, W.L. Gore & Associates, Inc. Medical Products Division, Flagstaff, Arizona, USA). All patients received a single shot of antibiotic prophylaxis and in most cases vancomycin 1 g was administered 30 min prior to skin incision. Of the prostheses 14.0 % (61/436) had to be explanted for several reasons: three for shunt aneurysms, two for large hematomas, one after renal transplantation and one for acute rupture. The remaining 54 shunt grafts were explanted due to infection. Nearly half of the infections developed in the first year (Fig. 1) but explantation was even peformed 8, 10 and 14 years after surgery with shunt grafts that had not been used for years.

In 61.1 % (33/54) of cases, monobacterial cultures could be found (Table 1) and in 13.0 % (7/54) 2 different bacteria were detected. In 3.7 % (2/54), 4 different bacteria were identified and in 22.2 % (12/54) bacterial cultures stayed sterile. *S. aureus* was present in 76.2 % (32/42) of cases with detected bacteria and nearly all isolates were resistant to penicillin and ampicillin susceptibility and susceptible to all other tested antibiotics (Table 2). None of the isolates were methicillin-resistant *S. aureus *(MRSA).

**Table 1:** Bacterial spectrum of 54 shunt graft infections at the Department of Surgery, Division of Vascular Surgery, MUG, Austria

*n*	54
Samples with detection of monocultures infections with one strain	33
*Staphylococcus aureus*	26
Coagulase negative *Staphylococcus* (CNS)	3
*Enterococcus* spp.	2
*Pseudomonas aeruginosa*	1
*Staphylococcus epidermidis*	1
Samples with co-infections with two strains	7
*Staphylococcus aureus* and *Enterococcus*	2
Coagulase negative *Staphylococcus, Staphylococcus epidermidis*	1
*Staphylococcus aureus, Staphylococcus epidermidis*	3
*Corynebacterium* spp., *Micrococcus* sp	1
Samples with polymicrobial infections with four strains	2
Coagulase negative *Staphylococcus, Staphylococcus epidermidis, Corynebact*., *Klebsiellia pneumoniae* ESBL	1
*Staphylococcus aureus, Enterococcus, Staphylococcus epidermidis, Klebsiellia oxytoca*	1
Infections without strain detection	12

**Table 2:** Antibiogram of most *S. aureus* cases

	Susceptibility
Ampicillin	R
Vancomycin	S
Teicoplanin	S
Quinupristin/dalfopristin	S
Linezolid	S
Nitrofurantoin	S
Cefazolin	S
Cefuroxime	S
Penicillin	R
Amoxicillin/clavulanic acid	S
Cefotaxime	S
Clarithromycin	S
Clindamycin	S
Moxifloxacin	S
Ceftriaxone	S
Piperacillin/tazobactam	S
Oxacillin	S
Gentamicin	S
Rifampicin	S
Fusidic acid	S
Fosfomycin	S
Trimethoprim	S
Meropenem	S
Cefpirom	S

Susceptibility of detected *S. aureus* to antibiotics *R*: resistant, *S*: susceptible

A total of 3 *S. aureus* strains from 2014 underwent *spa typing*. Three different types were found (worldwide frequency according to Ridom SpaServer): t015 (1.12 %), t359 (0.17 %) and t6265 (<0.01 %).

We investigated influencing factors (Table 3) but did not find any to be significant. The “dialysis institute” factor had a remarkable p‑value of 0.064. In one of them, the shunt graft explantation rate due to infection was 18.2 %, whereas it was 8.5 % and 9.6 % for the others.

**Table 3:** Impact of independent influencing factors on shunt graft infection rates

Independent factor	*p*-value
Sex	0.660
Institute for dialysis	0.064
Age	0.376
Inner coating of the PTFE prosthesis (none, carbon, heparin)	0.708
Implantation surgeon	0.810
Shunt position (upper extremity vs. lower)	0.537

## Discussion and conclusion

The primary goal of our study was to clarify whether there is a nosocomial problem. Spa typing of 3 cases from 2014 revealed 3 different genetic types, so they must have arisen from different sources and therefore a nosocomial problem is very unlikely. Spa typing is a typing method with a very high discriminatory index of 0.87 comparable to repetitive sequence-based PCR (0.88) and multilocus sequence typing (0.84). It is better than pulsed-field gel electrophoresis (0.76) and staphylococcal cassette chromosome mec typing (0.60) [14]. Spa typing therefore seems to be a good way to discriminate between nosocomial and community-acquired shunt graft infections.

For the chronological course of shunt graft explantations (Fig. 1), an exponential curve can easily be adjusted which is typical for nearly every biological process. If our shunt graft infections were a problem caused by the primary operation, nearly all explantations should have occurred in the first year; therefore we assume that the shunt graft patients were *S. aureus* carriers and furthermore that every *S. aureus* carrier will experience shunt graft infections over time just as a matter of chance. This must be proven in a separate trial.

In hemodialysis patients, nasal carriage of *S. aureus* is a risk factor for the development of *S. aureus* bacteremia. Nasal mupirocin ointment can be used to successfully eradicate *S. aureus* from nasal carriage and is able to reduce the incidence of *S. aureus* bacteremia by 75.25 % [15–17]; therefore we will implement nasal eradication of *S. aureus* as part of our standard procedures and monitor the results thoroughly. On the other hand, there were remarkable differences in shunt infection rates between the institutions which performed dialysis. We have contacted the institution with the unexpectedly high rate but have not yet found a reason for the difference. We assume different disinfection techniques to be responsible for that phenomenon but have not yet been able to prove it. In 22.2 % of our cases we did not detect any strain. This might be due to the fact that in cases of suspected infection, antibiotic treatment is induced immediately. Bacterial cultures from blood or the surgical wound might remain sterile if the time interval between induction of antibiotic treatment and surgery is long enough. Nevertheless, bacteria are still present in the biofilm of the prosthesis. In recent years we have therefore changed our strategy and always send a part of the prosthesis for microbiological investigation. We can recommend this technique most notably if the tissue around the prosthesis looks unsuspicious and antibiotic treatment has been started some days previously.
